# Supplementary material for: Basic β-1,3-Glucanase from Drosera binata Exhibits Antifungal Potential in Transgenic Tobacco Plants
Source: Plants (Basel). 2021 Aug 23;10(8):1747. doi: 10.3390/plants10081747 (PMC8401921; doi:10.3390/plants10081747)
Supplement: Supplementary file 1 [file plants-10-01747-s001.zip › plants-1332183-supplementary.pdf]

Supplementary Materials

Supplementary Table S1. The list of primers used in PCRs at bacterial and plant expression vector construction, and analysis of transgenic tobacco plants

Primer	Sequence (5′3′)	Expected size (bp)	Purpose
P1*<br>P2*	TTCACGTGATCGGGACATGCTATGGCATGCTA<br>TACACGTGGTTGAAGTTAACCGGATACTT	955	cDNA *DbGluc1* gene without signal peptide for expression in *E. coli*
P3<br>P4	ACCATGGCCCTCACACTTA<br>TCGTCTAGACTCAGTGATGGTGATGGTGATGG TTGAAGTTAACCGGATACTT	1132	Amplification of *DbGluc1* gene with His-tag
P5<br>P6	ATGACTGGGCACAACAGA<br>ARGGGTCACGACGAGATC	500	Detection of *nptII* gene
P7<br>P8	AACTTGGACAATGGAGCTTCT<br>ACTTCAGGTTCTCATTGTACA	580	Detection of *DbGluc1* gene
P9<br>P10	CACCACCACTGCAGAACGGGAAAT<br>AACGGAGGAGCTGCTCTTGGCA	109	*Actin* qPCR<br>*Actin* RT-qPCR
P11<br>P12	TACCTAAAACAGCTGATGCGATCGG<br>GGACCGGACCGTAGATTCTCATTCC	129	*DbGluc1* qPCR;<br>*DbGluc1* RT-qPCR

* underlined are sequences for *Pml*I restriction enzyme

Supplementary Table S2 Summary results of filamentous fungi growth in the presence of purified β-1,3-glucanase assayed spectrophotometrically

Fungi	Purified protein	Growth of pathogenic fungi (A_{595})												Average	Growth [%]	Significance ($p < 0.05$)
Trichoderma viride	Heat-treated protein	0.214	0.223	0.24	0.234	0.237	0.243	0.264	0.268	0.246	0.248	0.243	0.225	0.240	99.8	a
	Active protein	0.213	0.221	0.221	0.258	0.261	0.25	0.247	0.255	0.24	0.241	0.233	0.238	0.240		
Fusarium poae	Heat-treated protein	0.269	0.28	0.283	0.284	0.265	0.268	0.281	0.292	0.278	0.268	0.255	0.246	0.272	75.9	b
	Active protein	0.215	0.223	0.214	0.224	0.227	0.226	0.197	0.209	0.204	0.174	0.185	0.183	0.207		
Alternaria solani	Heat-treated protein	0.35	0.337	0.337	0.328	0.339	0.337	0.414	0.41	0.411	0.422	0.428	0.428	0.378	85.6	b
	Active protein	0.292	0.292	0.282	0.264	0.262	0.256	0.377	0.383	0.384	0.37	0.361	0.366	0.324		
Rhizoctonia solani	Heat-treated protein	0.252	0.255	0.263	0.281	0.281	0.276	0.322	0.314	0.316	0.28	0.286	0.286	0.284	82.5	b
	Active protein	0.251	0.248	0.24	0.207	0.214	0.213	0.25	0.257	0.253	0.231	0.229	0.222	0.235		

Supplementary Table S3. Transgene copy number estimation by qPCR in transgenic tobacco plants

Normalisator - transgenic line DD3 15

Transgenic line	Ratio of values	Copy number by Southern blotting
DD3 15	1	1
DD3 22	1.7	2

*tobacco actin (XM_016618658.1) was chosen as a reference gene

Normalisator - transgenic line 16

Transgenic line	Ratio of values	Estimated copy number
6	0.737	1
9	1.813	2
10	3.65	4
13	0.731	1
16	1	1
19	0.634	1
22	1.031	1
DD3 15	1.11	1
DD3 22	2.01	2

*tobacco actin (XM_016618658.1) was chosen as a reference gene

Supplementary Table S4. Supplementary results of the RT-qPCR used at analysis of relative expression levels of the *DbGluc1* gene in transgenic tobacco lines

Sample	Normalised expression replicate 1	Standard deviation replicate 1	Normalised expression replicate 2	Standard deviation replicate 2	Normalised expression replicate 3	Standard deviation replicate 3	Average normalised expression	Standard deviation of average
6	0.00492	0.00028	0.00506	0.00027	0.00419	0.00026	0.00472	0.00027
9	0.04623	0.00290	0.03639	0.00286	0.04032	0.00279	0.04098	0.00285
10	0.02459	0.00013	0.02504	0.00014	0.02464	0.00015	0.02476	0.00014
13	2.53768	0.25316	3.19409	0.24418	2.40387	0.23519	2.71188	0.24417
16	3.65342	0.08003	3.62753	0.07923	3.40386	0.07784	3.56160	0.07903
19	5.49058	0.24073	6.15542	0.20862	6.06833	0.17594	5.90478	0.20842
22	5.28041	0.27307	6.22388	0.27398	5.66279	0.27488	5.72236	0.27398

Supplementary Table S5. Supplementary results of the total β-1,3-glucanase activity assay in crude protein extracts of transgenic tobacco lines and the non-transgenic control

β-1,3-Glucanase		Absorbance					Enzyme activity			
		0 h	2 h				Activity	Relative	Average relative	Significant difference
Tobacco line	1 – top<br>2 – middle<br>3 – bottom		1 replicate	2 replicate	3 replicate	Average	[U/min]	[%]	[%]	$p < 0.05$
NT	1	0.317	0.489	0.514	0.547	0.517	0.669	**100.00**		a
	2	0.502	0.722	0.699	0.687	0.703	0.672	**100.00**	100.00	a
	3	0.756	0.978	0.924	0.97	0.957	0.674	**100.00**		a
6	1	0.446	0.637	0.659	0.681	0.659	0.713	**106.68**		a
	2	0.632	0.857	0.831	0.83	0.839	0.694	**103.32**	105.43	a
	3	0.801	1.012	1.036	0.997	1.015	0.717	**106.29**		a
9	1	0.459	0.682	0.691	0.693	0.689	0.769	**115.03**		b
	2	0.518	0.724	0.753	0.785	0.754	0.790	**117.61**	118.27	b
	3	0.758	0.932	1.007	1.073	1.004	0.824	**122.19**		b
10	1	0.424	0.647	0.633	0.671	0.650	0.758	**113.36**		b
	2	0.469	0.658	0.703	0.719	0.693	0.751	**111.79**	112.85	b
	3	0.829	1.012	1.074	1.086	1.057	0.765	**113.41**		b
13	1	0.328	0.72	0.712	0.696	0.709	1.277	**190.98**		c
	2	0.471	0.784	0.816	0.806	0.802	1.108	**164.95**	174.44	c
	3	0.739	1.202	1.005	1.021	1.076	1.128	**167.38**		c
16	1	0.423	0.758	0.773	0.755	0.762	1.135	**169.78**		c
	2	0.675	1.055	1.047	1.072	1.058	1.283	**190.86**	179.27	c
	3	0.896	1.259	1.227	1.272	1.253	1.194	**177.15**		c
19	1	0.428	0.944	0.921	0.936	0.934	1.693	**253.26**		c
	2	0.624	1.112	1.134	1.157	1.134	1.709	**254.32**	250.65	c

	3	0.699	1.18	1.194	1.199	1.191	1.648	**244.37**		c
22	1	0.438	0.932	0.926	0.933	0.930	1.649	**246.58**		c
	2	0.455	0.893	0.904	0.886	0.894	1.471	**218.94**	233.21	c
	3	0.807	1.292	1.256	1.287	1.278	1.578	**234.11**		c

1- Top, 2 – middle, 3 – bottom leaf ; % of activity; non-transgenic plant (NT) was considered as a plant with 100% enzyme activity

Supplementary Table S6. Summary results of antifungal activity effect of crude protein extracts isolated from transgenic tobacco lines and the non-transgenic control on the fungal growth of tested filamentous fungi

Fungi	Tobacco line	Growth of tested fungi (A_{595})															Average	Growth [%]	Significance ($p < 0.05$)
Trichoderma viride	NT	0.169	0.165	0.147	0.147	0.15	0.2	0.194	0.195	0.181	0.21	0.109	0.125	0.105	0.117	0.137	0.157	100.0	a
	6	0.26	0.201	0.185	0.211	0.046	0.217	0.197	0.208	0.191	0.203	0.098	0.097	0.094	0.093	0.106	0.160	102.4	a
	9	0.231	0.18	0.166	0.215	0.046	0.194	0.192	0.231	0.257	0.196	0.103	0.114	0.104	0.091	0.106	0.162	103.2	a
	10	0.148	0.191	0.156	0.172	0.046	0.207	0.207	0.201	0.224	0.225	0.096	0.1	0.093	0.096	0.09	0.150	95.8	a
	13	0.195	0.172	0.142	0.174	0.172	0.222	0.207	0.222	0.202	0.188	0.102	0.101	0.106	0.106	0.106	0.161	102.8	a
	16	0.132	0.13	0.147	0.161	0.136	0.211	0.214	0.249	0.207	0.186	0.095	0.127	0.089	0.103	0.093	0.152	97.0	a
	19	0.188	0.098	0.098	0.099	0.094	0.215	0.234	0.225	0.216	0.27	0.106	0.103	0.102	0.107	0.101	0.150	96.0	a
	22	0.093	0.092	0.111	0.1	0.171	0.236	0.22	0.221	0.271	0.225	0.108	0.107	0.099	0.099	0.174	0.155	99.0	a
Fusarium poae	NT	0.162	0.181	0.138	0.151	0.157	0.21	0.204	0.22	0.208	0.216	0.27	0.285	0.285	0.231	0.295	0.214	100.0	a
	6	0.253	0.213	0.195	0.23	0.046	0.263	0.255	0.258	0.222	0.22	0.231	0.216	0.223	0.476	0.188	0.233	108.6	a
	9	0.257	0.164	0.178	0.222	0.046	0.254	0.251	0.232	0.228	0.23	0.154	0.244	0.246	0.203	0.223	0.209	97.5	a
	10	0.178	0.152	0.201	0.186	0.046	0.232	0.216	0.246	0.219	0.227	0.19	0.095	0.091	0.092	0.092	0.164	96.3	a
	13	0.174	0.173	0.173	0.16	0.181	0.277	0.24	0.235	0.23	0.224	0.099	0.218	0.209	0.211	0.243	0.203	94.8	a
	16	0.139	0.125	0.146	0.13	0.136	0.223	0.237	0.227	0.213	0.274	0.245	0.191	0.181	0.218	0.163	0.190	88.6	a
	19	0.195	0.1	0.099	0.101	0.09	0.159	0.147	0.135	0.141	0.156	0.154	0.168	0.138	0.149	0.172	0.140	65.5	b
	22	0.092	0.093	0.109	0.104	0.155	0.156	0.14	0.283	0.201	0.149	0.165	0.162	0.154	0.177	0.165	0.154	71.7	b
Alternaria solani	NT	0.266	0.268	0.279	0.276	0.27	0.277	0.27	0.275	0.274	0.284	0.265	0.279	0.272	0.283	0.265	0.274	100.0	a
	6	0.271	0.262	0.271	0.263	0.268	0.263	0.27	0.264	0.261	0.272	0.261	0.266	0.267	0.263	0.274	0.266	97.4	a

	9	0.255	0.252	0.26	0.252	0.253	0.265	0.266	0.253	0.266	0.256	0.252	0.268	0.264	0.252	0.266	0.259	94.6	a
	10	0.269	0.263	0.27	0.264	0.259	0.265	0.257	0.266	0.27	0.258	0.26	0.261	0.263	0.269	0.271	0.264	96.6	a
	13	0.272	0.263	0.262	0.259	0.27	0.261	0.257	0.262	0.261	0.261	0.27	0.268	0.272	0.272	0.26	0.265	96.8	a
	16	0.277	0.256	0.275	0.278	0.265	0.259	0.252	0.267	0.277	0.254	0.276	0.262	0.252	0.272	0.253	0.265	96.9	a
	19	0.234	0.237	0.24	0.242	0.242	0.235	0.235	0.239	0.234	0.228	0.23	0.228	0.233	0.234	0.238	0.235	86.0	b
	22	0.236	0.237	0.233	0.239	0.233	0.241	0.251	0.24	0.237	0.234	0.233	0.232	0.238	0.234	0.233	0.237	86.5	b
Rhizoctonia solani	NT	0.252	0.261	0.265	0.263	0.258	0.267	0.26	0.265	0.257	0.249	0.263	0.258	0.252	0.257	0.255	0.259	100.0	a
	6	0.269	0.269	0.254	0.264	0.254	0.257	0.269	0.269	0.252	0.258	0.267	0.26	0.261	0.257	0.253	0.261	100.8	a
	9	0.256	0.258	0.261	0.25	0.261	0.259	0.262	0.259	0.242	0.248	0.255	0.257	0.25	0.251	0.244	0.254	98.2	a
	10	0.261	0.245	0.242	0.256	0.263	0.252	0.261	0.256	0.246	0.26	0.258	0.26	0.258	0.258	0.25	0.255	98.6	a
	13	0.245	0.253	0.243	0.255	0.243	0.247	0.26	0.256	0.261	0.263	0.242	0.249	0.248	0.256	0.26	0.252	97.4	a
	16	0.261	0.262	0.257	0.262	0.262	0.247	0.253	0.262	0.25	0.243	0.262	0.247	0.256	0.253	0.245	0.255	98.5	a
	19	0.219	0.217	0.223	0.209	0.216	0.218	0.22	0.216	0.211	0.205	0.216	0.216	0.209	0.223	0.211	0.215	83.2	b
	22	0.208	0.214	0.206	0.22	0.213	0.223	0.215	0.216	0.22	0.207	0.214	0.215	0.223	0.219	0.209	0.215	83.0	b

Supplementary Table S7. Supplementary results of the total chitinolytic activity assay in crude protein extracts of transgenic tobacco lines and the non-transgenic control

Chitinase	Absorbance						Enzyme activity			
		0 h	2 h				Activity	Relative	Average relative	Significant difference
Tobacco line	1 – top<br>2 – middle<br>3 – bottom		1 replicate	2 replicate	3 replicate	Average	[U/min]	[%]	[%]	$p < 0.05$
NT	1	0.286	0.465	0.483	0.512	0.487	0.742	**100.00**	a	
	2	0.282	0.479	0.465	0.494	0.479	0.730	**100.00**	a	100.00
	3	0.269	0.483	0.462	0.493	0.479	0.778	**100.00**	a	
6	1	0.242	0.456	0.433	0.45	0.446	0.756	**101.83**	a	
	2	0.286	0.502	0.509	0.488	0.500	0.790	**108.28**	a	105.48
	3	0.255	0.478	0.476	0.482	0.479	0.827	**106.34**	a	
9	1	0.274	0.452	0.478	0.466	0.465	0.707	**95.35**	a	
	2	0.291	0.512	0.484	0.485	0.494	0.749	**102.70**	a	100.62
	3	0.284	0.506	0.508	0.493	0.502	0.807	**103.80**	a	
10	1	0.26	0.449	0.443	0.459	0.450	0.704	**94.85**	a	
	2	0.248	0.424	0.465	0.459	0.449	0.744	**102.03**	a	98.06
	3	0.266	0.457	0.483	0.472	0.471	0.757	**97.31**	a	
13	1	0.278	0.477	0.495	0.503	0.492	0.790	**106.48**	a	
	2	0.254	0.447	0.465	0.442	0.451	0.730	**100.00**	a	104.54
	3	0.284	0.503	0.509	0.516	0.509	0.833	**107.13**	a	
16	1	0.309	0.529	0.513	0.475	0.506	0.727	**98.01**	a	
	2	0.283	0.449	0.491	0.482	0.474	0.706	**96.79**	a	96.21
	3	0.262	0.462	0.459	0.457	0.459	0.730	**93.82**	a	
19	1	0.291	0.523	0.509	0.531	0.521	0.850	**114.62**	b	
	2	0.241	0.485	0.489	0.486	0.487	0.908	**124.49**	b	121.86

	3	0.244	0.508	0.503	0.519	0.510	0.984	**126.47**	b	
22	1	0.271	0.462	0.519	0.534	0.505	0.865	**116.61**	b	
	2	0.23	0.483	0.441	0.469	0.464	0.866	**118.75**	b	120.66
	3	0.219	0.501	0.471	0.484	0.485	0.985	**126.62**	b	

1- Top, 2 – middle, 3 – bottom leaf; % of activity; non-transgenic plant (NT) was considered as a plant with 100% enzyme activity
